# How to determine if a model is right for neglected tropical disease decision making

**DOI:** 10.1371/journal.pntd.0005457

**Published:** 2017-04-20

**Authors:** Bruce Y. Lee, Sarah M. Bartsch

**Affiliations:** 1 Public Health Computational and Operations Research, Johns Hopkins Bloomberg School of Public Health, Baltimore, Maryland, United States of America; 2 Global Obesity Prevention Center, Johns Hopkins University, Baltimore, Maryland, United States of America; Yale School of Public Health, UNITED STATES

## Abstract

Mathematical and computational modeling can transform decision making for neglected tropical diseases (NTDs) if the right model is used for the right question. Modeling can help better understand and address the complex systems involved in making decisions for NTD prevention and control. However, all models, modelers, and modeling are not the same. Thus, decision makers need to better understand if a particular model actually fits their needs. Here are a series of questions that a decision maker can ask when determining whether a model is right for him or her.

Mathematical and computational modeling can transform decision making for neglected tropical diseases (NTDs) if it is the right model for the right question. Modeling has revolutionized decision making in many other fields such as meteorology, manufacturing, transportation, and air traffic control. For example, prior to the advent of simulation models that could generate weather maps, weather forecasting and planning had to rely on much more haphazard observations. Modeling can help better understand and address the complex systems (e.g., biological, behavioral, environmental, cultural, and economic) involved in making decisions for NTD prevention and control, which unaided can be challenging. Fig 1 shows the timeline/life cycle for a product or policy and how modeling can aid decision making at each step. However, all models, modelers, and modeling are not the same. Models range from those primarily oriented towards scientific inquiry to those that assist decision making with many variations in between. Therefore, decision makers need to better understand if a model actually fits their needs. What follows are the questions that a decision maker can ask when determining whether a model is right for him or her.

**Fig 1:**
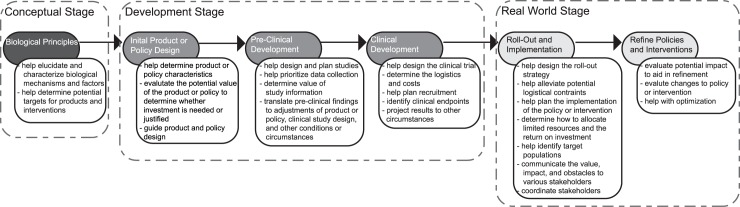
Examples of how modeling can help decision making at each stage in the product and policy development and implementation timeline.

## Question 1: Does the modeler understand me?

In any business, understanding the client is paramount to designing and providing a product or service. Similarly, without understanding the decision maker, there is risk of developing a model that does not fit or capture the needs of the decision maker. These needs can be complex, subtle, and not apparent to a modeler who is unable to place himself or herself in the shoes of the decision maker. Understanding the decision maker requires humility, a desire to listen and learn, and preferably direct experience. Nothing can really replace experience. However, without true experience, the modeler has to put in considerable extra work to truly relate. For example, a common complaint among physicians and policy makers is that a model does not accurately represent the realistic options and constraints that they face. This raises the second question.

## Question 2: Does the model reflect my real-world constraints?

While models are simplifications of the real world, to be useful to a decision maker, the model needs to include the constraints that the decision maker may face. This is a key distinction between models that are designed more for pure scientific inquiry and those that aim to assist decision making. Common real-world constraints include economics (e.g., some interventions may be cost prohibitive), logistics (e.g., getting medications to a population requires functional and far-reaching supply chains) and behavior (e.g., people may not comply with completing a course of medications).

## Question 3: Does the model incorporate all the considerations?

Models for decision making are by definition, multi- and inter-disciplinary. A decision is rarely specific to a discipline. In other words, a decision is not purely a biology decision, an epidemiology decision, or an economic decision. Instead, a decision typically spans multiple disciplines and considerations. There may be biological, epidemiological, or economic considerations that need to be integrated.

## Question 4: Was the model originally designed for this purpose?

Choosing the right model is important. All models are not the same. Instead, a tremendous diversity of models exist, and different models serve different purposes. For example, some modelers and models are more oriented towards specific biological or transmission processes and scientific inquiry. Others help understand and guide clinical processes. Still others focus more on policy making. No single model or modeler can do all of these or serve other similar disparate purposes at once. Similarly, a wide variety of modeling methodologies exist, ranging from decision analytic models, to compartment models, to network models, to agent-based models, and many other types and hybrids along the spectrum of possibilities. No single methodology is appropriate for all situations or necessarily superior or more complex than other methodologies. Repurposing a model that was originally designed for another purpose can be analogous to using a car as a boat.

## Question 5: Is the model too simple or too complex?

The principle of parsimony specifies that a model should not be more complex than needed. In other words, a model should only include what’s necessary. A model is not a replica (i.e., something that is supposed to mirror everything in real life), but instead a distillation of the important factors and relationships germane to a question or decision. For instance, detailed representations of pathogen transmission that do not significantly affect cost or effectiveness may not be necessary in an economic model. On the other extreme, models that oversimplify important processes or leave out key factors or elements may produce misleading results (e.g., a model that determines the value of a treatment but does not account for the costs and health effects).

## Question 6: Does the model take the appropriate perspective?

The modeler should clearly identify the perspective of the model, which in turn should match the perspective (i.e., viewpoint) of the decision maker. Different decision makers have different perspectives. For example, an employer may be interested in how a vaccine affects company costs and productivity, whereas an individual patient may be more interested in how a vaccine affects his or her own health and finances.[1–3] A third party payer (or insurance company) may focus on the direct medical costs incurred and saved by a vaccine, which represent what the third party must pay. The societal perspective accounts for both direct medical costs and productivity losses. Model results can differ considerably by perspective (e.g., the annual direct medical costs of Chagas disease may be US$474 but the societal costs are much higher at US$4,660[4]).

## Question 7: Does the model use the appropriate time frame?

The modeler should also clearly identify the time frame of the model. Time frame matters in decision making. The model should represent the duration of time that is relevant to decision making. A model that only represents a day after the administration of a vaccine would not capture its potential costs and benefits. For example, capturing the value of a vaccine with a 20-year duration of protection would require running a model for at least 20 years.[5]

## Question 8: Does the model require too much data?

Decision makers frequently have to make decisions under uncertainty and data sparse situations. A modeler asking for extensive amounts of data that require too much time to analyze can defeat a model’s utility. A common myth is that models are only as good as the data, i.e., garbage in, garbage out. Depending on the type and structure of the model, this is not necessarily true. Some models rely more heavily on data, such as those in which the modeler essentially fits mathematical equations onto specific datasets. However, other models attempt to represent different relevant mechanisms by other means besides extensive datasets. They can make certain assumptions as long as they are transparent and justifiable, draw learnings from other fields and settings, or develop representations based on observations and theories. Extensive sensitivity analyses can help test the robustness of assumptions and various representations. This is especially relevant for NTDs, for which data is often sparse, of poor quality, or absent. In fact, trying to fit models on poor-quality data can actually lead to misleading findings. The relative lack of data should not prevent the building of models. In fact, building models can help determine what data is needed and the value of additional data, which can guide and prioritize data collections.

## Question 9: Does the model generate relevant measures and outcomes?

Each type of decision maker tends to rely on particular measures to make decisions. For example, those with financial responsibilities will focus on financial measures. Health care workers will be very interested in key health outcomes, such as deaths, hospitalizations, and incidence of various types of diseases. Model outcomes should lead to actionable steps. For example, the reproductive rate (R0) may be of interest to an epidemiologist, but have little meaning to a policy maker. Additionally, modelers need to be able to generate graphs and visualizations that communicate findings in a concise and easily comprehendible manner. Many scientific methods of presenting data just do not resonate with decision makers.

## Question 10: Is the modeler transparent about what the model can and cannot do?

Just like every tool, product, or service, every model has its strengths and limitations. Misuse of models is common and is analogous to using a hammer as a saw. A model that is very good for a given purpose can be very bad for another. Ultimately, decision makers should be wary of any model that is a “black box” to them. Modelers need to be willing and able to effectively communicate their model’s structure and components, strengths and weaknesses, and associated assumptions and limitations. In many ways, sensitivity analyses (exploring the effects of varying key model parameters) are the most important model results, as they show how it performs under different conditions and circumstances. The analogy is experiencing how a car behaves during a test drive.

Table 1 shows examples of questions decision makers may ask and the models that have been used to answer such questions. While modeling can help address many current challenges of NTD decision making (e.g., complex systems impacted by environmental, economic, behavioral, and biologic factors[6]; subtle and indirect impacts that accumulate over time[7]), both modelers and decision makers have to better appreciate all of the above considerations so that they can choose the right model to fit the right decision.

**Table 1:** Examples of questions decision makers may ask and models that have answered such questions.

Example Questions	Example Models
How significant is an NTD, and how much time, effort, and resources should be dedicated to prevention and control?	Globally, human hookworm infection costs society US$7.5 billion to US$138.9 billion in 2016 and results in 4.1 million DALYs. The cost per hookworm infection varies by geographic region, from US$99 in Southeast Asia to US$447 in the Americas.[8]
What is the impact of an NTD on different locations, sectors, and industries?	Chagas disease cost US$627 million in health care costs and US$7 billion in societal costs. Annually, an infected individual costs US$4,059 in Latin America, US$13,580 in Europe, and US$15,762 in the United States, Canada, and Australia.[4]
What level of control (e.g., eradication, elimination, or long-term control) of the NTD is possible?	MDA for lymphatic filariasis would need to be drastically scaled up (85% coverage rate with all countries treating 100% of at-risk population annually) to achieve eradication. Maintaining current rates would mean eradiation would not be possible until 2050, with the last round of MDA being given then.[9]
Which existing policies, products, and strategies should be used to prevent and control an NTD?	A combination of treatment of infective individuals and insecticide spraying was the most effective and cost-effective (economically dominant) method of control for visceral leishmaniasis. Insecticide spraying would require less effort when used in combination with treatment policies.[10]
What new policies, products, and strategies for NTD prevention and control should be developed and what should be their characteristics?	A human hookworm vaccine would be more cost-effective than currently used MDA except when vaccination was less efficacious (20% efficacy, 5-year duration) and MDA coverage was 75%. The prevalence in children decreased to 14.6% after 20 years with vaccination, compared to 54.1% with MDA.[5]
How should NTD policies, products, or strategies be implemented?	Hookworm vaccination in combination with drug treatment would be cost-effective when vaccinating both school-aged children and women of child-bearing age but may yield a greater economic return among school-aged children, as the coverage may be greater in this population.[11]
How much should be invested in the development of new policies, products, and strategies for NTD prevention and control?	The return-on-investment for a therapeutic Chagas vaccine would reach US$2 million to US$18 million if 3% of the target population is vaccinated and development costs were $100 million or if 11% are vaccinated and development costs were $400 million.[12]
How should a population be screened for a NTD?	Screening Latin American pregnant women and their newborns for Chagas disease in nonendemic settings economically dominated (cost less and provided health benefits) no screening. Even at a low prevalence of Chagas (0.9%) with a low probability of vertical transmission (2.24%), screening was still better than no screening at a cost of €37.5 per test.[13]
What should be the price of a NTD product or service?	To cost less to avert a case than the cost of treatment, a vaccine for cutaneous leishmaniasis that is US$0.5 per dose (2 doses) would need to have an efficacy of 70% and 5-year protection duration with a ≥0.1% infection risk.[14]
Which of the available NTD policies, products, and strategies should be used?	Among four drug treatment regimens currently used for visceral leishmaniasis, amphotericin B deoxycholate was the most effective, averting 87.2% of attributable deaths, while miltefosine was the most cost-effective.[15]

DALY, disability-adjusted life year; MDA, mass drug administration; NTD, neglected tropical disease.

## References

[1] Lee BY, Bailey RR, Wiringa AE, Afriyie A, Wateska AR, Smith KJ, et al Economics of employer-sponsored workplace vaccination to prevent pandemic and seasonal influenza. Vaccine. 2010;28:5952–9. doi: 10.1016/j.vaccine.2010.07.003 20620168 10.1016/j.vaccine.2010.07.003 PMC2926133

[2] Lee BY, Brown ST, Cooley PC, Zimmerman RK, Wheaton WD, Zimmer SM, et al A computer simulation of employee vaccination to mitigate an influenza epidemic. American Journal of Preventive Medicine. 2010;38(3):247–57. doi: 10.1016/j.amepre.2009.11.009 20042311 10.1016/j.amepre.2009.11.009 PMC2833347

[3] Lee BY, Bacon KM, Donohue JM, Wiringa AE, Bailey RR, Zimmerman RK. From the patient perspective: the economic value of seasonal and H1N1 influenza vaccination. Vaccine. 2011;29:2149–58. doi: 10.1016/j.vaccine.2010.12.078 21215340 10.1016/j.vaccine.2010.12.078 PMC3046420

[4] Lee BY, Bacon KM, Bottazzi ME, Hotez PJ. Global economic burden of Chagas disease: a computational simulation model. Lancet Infect Dis. 2013;13(4):342–8. Epub 2013/02/12. doi: 10.1016/S1473-3099(13)70002-1 23395248 10.1016/S1473-3099(13)70002-1 PMC3763184

[5] Bartsch SM, Hotez PJ, Hertenstein DL, Diemert DJ, Zapf KM, Bottazzi ME, et al Modeling the economic and epidemiologic impact of hookworm vaccine and mass drug administration (MDA) in Brazil, a high transmission setting. Vaccine. 2016;34(19):2197–206. doi: 10.1016/j.vaccine.2016.03.018 27002501 10.1016/j.vaccine.2016.03.018 PMC5547742

[6] Aagaard-Hansen J, Chaignat C-L. Neglected tropical diseases: equity and social determinants Equity, Social Determinants and Public Health Programmes. Geneva, Switzaerland: World Health Organization (WHO); 2010.

[7] Molyneux D. Neglected tropical diseases. Community Eye Health. 2013;26(82):21–4. PubMed PMID: 24023397; PubMed Central PMCID: PMCPMC3756642. 24023397 PMC3756642

[8] Bartsch SM, Hotez PJ, Asti L, Zapf KM, Bottazzi ME, Diemert DJ, et al The Global Economic and Health Burden of Human Hookworm Infection. PLoS Negl Trop Dis. 2016;10(9):e0004922 PubMed Central PMCID: PMCPMC5015833. doi: 10.1371/journal.pntd.0004922 27607360 10.1371/journal.pntd.0004922 PMC5015833

[9] Kastner RJ, Stone CM, Steinmann P, Tanner M, Tediosi F. What Is Needed to Eradicate Lymphatic Filariasis? A Model-Based Assessment on the Impact of Scaling Up Mass Drug Administration Programs. PLoS Negl Trop Dis. 2015;9(10):e0004147 PubMed Central PMCID: PMCPMC4599939. doi: 10.1371/journal.pntd.0004147 26451729 10.1371/journal.pntd.0004147 PMC4599939

[10] Biswas S, Subramanian A, IM EL, Chattopadhyay J, Sarkar RR. Optimal combinations of control strategies and cost-effective analysis for visceral leishmaniasis disease transmission. PLoS ONE. 2017;12(2):e0172465 doi: 10.1371/journal.pone.0172465 28222162 10.1371/journal.pone.0172465 PMC5319670

[11] Lee BY, Bacon KM, Bailey RR, Wiringa AE, Smith KJ. The potential economic value of a hookworm vaccine. Vaccine. 2011;29:1201–10. doi: 10.1016/j.vaccine.2010.12.004 21167860 10.1016/j.vaccine.2010.12.004 PMC3038553

[12] Lee BY, Bacon KM, Wateska AR, Bottazzi ME, Dumonteil E, Hotez PJ. Modeling the economic value of a Chagas' disease therapeutic vaccine. Hum Vaccin Immunother. 2012;8(9):1293–301. Epub 2012/08/17. PubMed Central PMCID: PMC3579910. doi: 10.4161/hv.20966 22894964 10.4161/hv.20966 PMC3579910

[13] Sicuri E, Munoz J, Pinazo MJ, Posada E, Sanchez J, Alonso PL, et al Economic evaluation of Chagas disease screening of pregnant Latin American women and of their infants in a non endemic area. Acta Trop. 2011;118(2):110–7. Epub 2011/03/15. doi: 10.1016/j.actatropica.2011.02.012 21396345 10.1016/j.actatropica.2011.02.012

[14] Bacon KM, Hotez PJ, Kruchten SD, Kamhawi S, Bottazzi ME, Valenzuela JG, et al The potential economic value of a cutaneous leishmaniasis vaccine in seven endemic countries in the Americas. Vaccine. 2013;31(3):480–6. PubMed Central PMCID: PMC3763201. doi: 10.1016/j.vaccine.2012.11.032 23176979 10.1016/j.vaccine.2012.11.032 PMC3763201

[15] Vanlerberghe V, Diap G, Guerin PJ, Meheus F, Gerstl S, Van der Stuyft P, et al Drug policy for visceral leishmaniasis: a cost-effectiveness analysis. Trop Med Int Health. 2007;12(2):274–83. Epub 2007/02/16. doi: 10.1111/j.1365-3156.2006.01782.x 17300636 10.1111/j.1365-3156.2006.01782.x

